# Combined Use of Sleep Quality and Duration Is More Closely Associated With Mortality Risk Among Older Adults: A Population-based Kyoto-Kameoka Prospective Cohort Study

**DOI:** 10.2188/jea.JE20220215

**Published:** 2023-12-05

**Authors:** Daiki Watanabe, Tsukasa Yoshida, Yuya Watanabe, Yosuke Yamada, Motohiko Miyachi, Misaka Kimura, the Kyoto-Kameoka Study Group

**Affiliations:** 1 Faculty of Sport Sciences, Waseda University, Saitama, Japan; 2 National Institute of Health and Nutrition, National Institutes of Biomedical Innovation, Health and Nutrition, Tokyo, Japan; 3 Institute for Active Health, Kyoto University of Advanced Science, Kyoto, Japan; 4 Senior Citizen’s Welfare Section, Kameoka City Government, Kyoto, Japan; 5 Physical Fitness Research Institute, Meiji Yasuda Life Foundation of Health and Welfare, Tokyo, Japan; 6 Department of Nursing, Doshisha Women’s College of Liberal Arts, Kyoto, Japan; 7 Laboratory of Applied Health Sciences, Kyoto Prefectural University of Medicine, Kyoto, Japan

**Keywords:** sleep quality, sleep duration, interaction, older adults, dose-response relationship

## Abstract

**Background:**

Whether sleep quality and duration assessed from multiple domains, either individually or in combination, are strongly associated with mortality risk in older adults remains unelucidated. We aimed to clarify these relationships.

**Methods:**

We enrolled 7,668 older (age ≥65 years) Japanese adults in the Kyoto-Kameoka prospective cohort study who provided valid responses to the Pittsburgh Sleep Quality Index (PSQI) in a mail-in survey. Sleep quality and duration were classified into six groups using the previously validated PSQI: short sleep duration (SSD: <360 min/day)/sleep disturbance (SD: ≥5.5 PSQI points), *n* = 701; SSD/non-sleep disturbance (NSD: <5.5 PSQI points), *n* = 100; optimal sleep duration (OSD: 360–480 min/day)/NSD, *n* = 1,863; OSD/SD, *n* = 2,113; long sleep duration (LSD: >480 min/day)/NSD, *n* = 1,972; LSD/SD, *n* = 919. Mortality data were collected from February 15, 2012, to November 30, 2016. We evaluated the relationship between all-cause mortality risk and sleep quality and duration (and their combinations) using a multivariable Cox proportional hazards model that included baseline covariates.

**Results:**

The median follow-up period was 4.75 years (34,826 person-years), with a total of 616 deaths. After adjusting for confounders, compared with other groups, SSD/SD and LSD/SD had the highest hazard ratio (HR) of mortality (SSD/SD: HR 1.56; 95% confidence interval [CI], 1.10–2.19; SSD/NSD: HR 1.27; 95% CI, 0.47–3.48; OSD/NSD: reference; OSD/SD: HR 1.20; 95% CI, 0.91–1.59; LSD/NSD: HR 1.35; 95% CI, 1.03–1.77; LSD/SD: HR 1.83; 95% CI, 1.37–2.45). However, mortality risk was not associated with the interaction between sleep quality and duration.

**Conclusion:**

Older adults with sleep disturbances involving SSD and LSD have a strong positive association with mortality risk, suggesting an additive effect between sleep quality and duration.

## INTRODUCTION

Sleep disorders include potentially overlapping symptoms and disorders, such as insomnia, hypersomnia, excessive daytime sleepiness, circadian rhythm disorders, and exogenous sleep disorders (poor sleep environment).^1^ They often associate with neuropsychological disorders^2^ and suicide risk.^3^ Approximately 50% of older adults experience sleep disorders,^2^ and resolving sleep problems could improve quality of life and daytime functions.^4^ Consequently, it is important to evaluate the impact of sleep status in older adults.

The concept of sleep health includes individual sleep-related symptoms and disorders and a holistic framework that includes multiple sleep domains (sleep duration, continuity, timing, awakening, and satisfaction).^5^ Many self-reported questionnaires have been developed to measure various aspects of sleep disorders.^6^ The Pittsburgh Sleep Quality Index (PSQI)^7^ was designed to assess sleep quality from multiple domains, rather than to screen for specific sleep disorders.^8^ This allows for evaluation of multidimensional sleep health as a continuous variable that characterizes all individuals in a population.^5^ This provides a higher quality comprehensive sleep status risk assessment than assessment comprising a single domain.

A meta-analysis^9^ and pooled analysis^10^ evaluating relationships between sleep duration and mortality risk reported that individuals with approximately 7 hours of sleep had the lowest risk of death. When assessed using a single domain, the combination of sleep duration and sleep disorders showed a stronger association with mortality risk than when assessed with either of these statuses.^11^^,^^12^ In a young and middle-aged Dutch cohort, those with poor sleep quality assessed using multidimensional sleep symptoms and with short sleep duration (SSD) had the highest risk of developing cardiovascular disease (CVD).^13^ To the best of our knowledge, the combination of sleep quality and duration, when evaluated from a multidimensional domain related to mortality risk in older adults aged ≥65 years, is insufficiently investigated.^14^ This study aimed to investigate the relationship between sleep quality and duration combinations (assessed using a previously validated PSQI) and all-cause mortality in a community-based longitudinal cohort study of older adults. As previous studies demonstrated, a U-shaped relationship exists between sleep duration and mortality.^9^^,^^10^ We hypothesized that older adults with both SSD and long sleep duration (LSD) with sleep disturbances (SD) would most strongly show association with all-cause mortality.

## METHODS

### Study design

**Figure 1.**
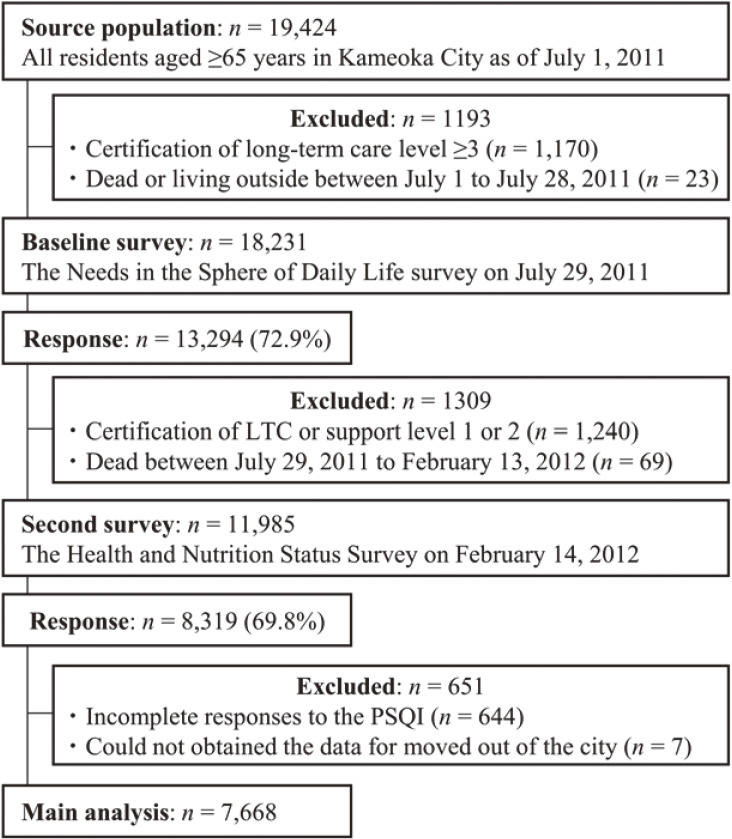
Participant flow diagram for the analysis of sleep quality and duration and mortality in Kyoto-Kameoka study. PSQI, Pittsburgh Sleep Quality Index; LTC, long-term care.

The Kyoto-Kameoka study is a prospective cohort study of older adults aged ≥65 years who lived in Kameoka City, Kyoto Prefecture, Japan. The study details are described elsewhere.^15^^–^^18^ To survey all residents of Kameoka aged ≥65 years as of July 1, 2011, qualified candidates were selected from the basic residents’ registry, managed by Kameoka City Hall (Figure 1). The Needs in the Sphere of Daily Life survey (baseline survey) was conducted by mail on July 29, 2011, for 18,231 residents after excluding those needing nursing care (*n* = 1,170) or who died between July 1 and 28, 2011 (*n* = 23). Of 18,231 residents, 13,294 responded to the survey (response rate: 72.9%). Health-related information, including medical history, socioeconomic status, smoking status, and alcohol intake, were obtained. Next, on February 14, 2012, the Health and Nutrition Status Survey (additional survey), which included the PSQI to assess sleep quality and duration, was conducted on 11,985 residents. This survey received validated responses from 8,319 residents (response rate: 69.8%). Of 8,319 residents, we excluded those with incomplete PSQI response (*n* = 644) and those with missing date of when they moved into/out of Kameoka City (*n* = 7), including 7,668 participants in the present study.

This study’s protocol was approved by the ethics committees of the National Institutes of Biomedical Innovation, Health, and Nutrition (NIBIOHN-76-2), Kyoto University of Advanced Science (No. 20-1), and Kyoto Prefectural University of Medicine (RBMR-E-363). Informed consent was obtained from all participants at the time of their response to the mail survey. Reporting for this study followed the Strengthening the Reporting of Observational Studies in Epidemiology.^19^

### Evaluation of sleep quality and duration

We evaluated the quality and duration of sleep over the preceding 1 month using PSQI,^7^ an 18-item questionnaire for assessing sleep quality developed by Buysse et al.^8^ We asked participants to complete the relevant number of questions about bedtime, fall asleep time, wake-up time, and sleep duration and to choose an appropriate number on a 4-point Likert scale (0–3 points) for other questions. The PSQI has seven subdomains related to sleep (C1: sleep quality, C2: sleep latency, C3: sleep duration, C4: habitual sleep efficiency, C5: sleep disturbances, C6: medications use to sleep, and C7: daytime dysfunction). The subdomain scores were totaled to calculate the Global PSQI score, which ranges from 0 (best sleep quality) to 21 points (worst sleep quality). The SD based on the PSQI, was defined as a score ≥5.5 out of 21.^20^ In previous Japanese studies, a cut-off PSQI score of 5.5 points predicted primary insomnia (sensitivity, 85.7%; specificity, 86.6%).^20^ This is comparable to the accuracy of predicting poor sleepers with PSQI scores reported by Buysse et al (sensitivity, 89.6%; specificity, 86.5%).^8^

We assessed individuals’ sleep duration (hours and minutes) using the following a question included in PSQI: “During the past month, how many hours of actual sleep did you get at night?” (This may be different than the number of hours you spend in bed). Sleep duration was classified into the following three groups, as previously reported^21^: SSD, <360 min/day; optimal sleep duration (OSD), 360–480 min/day; and LSD, >480 min/day.

### Outcomes

The participants’ survival status during the follow-up period was assessed using the resident’s basic registry information from February 15, 2012, to November 30, 2016. We censored ex-officio residents and those who left the country or moved.

### Statistical analysis

Participants were classified into the following six groups according to sleep quality and duration: SSD/SD, *n* = 701; SSD/non-sleep disturbance (NSD), *n* = 100; OSD/NSD, *n* = 1,863; OSD/SD, *n* = 2,113; LSD/NSD, *n* = 1,972; and LSD/SD, *n* = 919.

Descriptive statistics for continuous variables are expressed as mean and standard deviation and, for categorical variables, as number and percentages. Missing covariate values were imputed from five datasets created through multiple imputation using the multivariate imputation by chained equation package in the R statistical software (R Foundation for Statistical Computing, Vienna, Austria).^22^ All missing values were assumed to be missing at random. Further, to evaluate the reproducibility of the self-reported sleep duration assessments, we calculated intraclass correlation coefficients (ICC) between sleep duration estimated from the questions in the PSQI and the sleep duration data from the baseline survey.

The absolute risk of all-cause mortality for each of the six groups based on sleep quality and duration was expressed as number of events per 1,000 person-years. To adjust for confounders of sleep quality and duration and all-cause mortality risk, we used a multivariable Cox proportional hazards model including baseline covariates. In these analyses, time scale was a survey time. The Schoenfeld residuals test was performed to confirm the assumptions of the Cox proportional-hazard model. Proportional hazards conditions were assumed because the test did not reject the data (*P* = 0.180). Multivariable analysis was performed as follows: model 1 was adjusted for age (continuous), sex (female or male), and population density (≥1,000 or <1,000 people/km^2^). Model 2 was adjusted for model 1 factors plus body mass index (continuous), living alone (yes or no), socioeconomic status (high or low), educational attainment (<9, 10–12, or ≥13 years), smoking status (never smoker, past smoker, or current smoker), alcohol drinker (yes or no), physical activity (continuous), medication use (continuous), and number of chronic diseases (continuous). These adjustment factors were determined based on previous research.^11^^–^^14^ The previous week’s physical activity was evaluated using the International Physical Activity Questionnaire-Short Form (IPAQ-SF). The physical activity estimated from IPAQ-SF has been validated against the physical activity estimated using an accelerometer in Japanese adults aged 65 years and older (Spearman’s rank correlation coefficient = 0.43–0.54).^23^ We asked the participants for only the number of medications used, but not the kinds of drugs. From the data obtained on disease status (including the presence of hypertension, stroke, heart disease, diabetes, hyperlipidemia, digestive disease, respiratory disease, urological diseases, and cancer), the number of chronic diseases (comorbidity scores) were summed to obtain a total score ranging from 0 (no comorbidity) to 9 (poor status).^15^ The results are expressed as hazard ratios (HRs) and 95% confidence intervals (CIs). HRs were calculated based on the OSD/NSD group as reference. Interaction assessment between outcome and exposure is best presented using both additive and multiplicative interaction measurements.^24^ Therefore, we calculated both additive interaction (relative excess risk due to interaction: RERI) and multiplicative interaction using categorical variables for sleep quality and duration.

To rule out the possibility of reverse causality, we performed sensitivity analyses according to the following three methods: 1) we excluded death events (135 men, 65 women) in the first 2 years of follow-up; 2) we excluded participants with a history of CVD and cancer; 3) we performed a similar analysis using data on sleep disturbance and tertile of sleep duration for higher statistical power using the same sample size. We also created Nelson-Aalen cumulative hazard curves of mortality based on sleep quality and duration status, using age as time scale.^25^ To assess the relationship curve of sleep quality and duration with all-cause mortality risk, we used a restricted cubic spline model with three-point data based on the distributions of these values.^15^^,^^17^^,^^18^ These results are expressed as HR and 95% CI, with HR calculated based on sleep quality (0 points of PSQI) or sleep duration (420 min/day).^9^^,^^10^ We also calculated the HR and 95% CI for mortality risk in each sleep duration group if the six PSQI subdomain scores (excluding the sleep duration subdomain) increased by 1 point. The *P*-value of the linear trend was calculated by treating the exposure variable as a continuous variable.

A two-tailed significance level of <5% was used in the analysis. All analyses were performed using STATA MP, version 15.0 (StataCorp LP, College Station, TX, USA) and/or R software 3.4.3.

## RESULTS

### Demographics

Table 1 shows the participant characteristics by group based on sleep quality and duration. Compared to the OSD/NSD group, the LSD/SD group was older, had more diseases, and had fewer individuals in high economic status, not taking medication, and with higher educational attainment, while SSD/SD group had more women and more diseases. In addition, participants in this study were younger, had a higher education attainment, and had a higher economic status compared with those in the baseline survey (eTable 1). The prevalence of SDs was 48.7% (95% CI, 47.6–49.8) (eTable 2). When the sample was stratified by age, sex, sleep duration, area, and midpoint sleep (median point between bedtime and wake-up time), higher prevalence of SDs was observed among women, those aged ≥75 years, those who slept <360 min/day, rural residents (<1,000 people/km^2^), and those whose chronotype was night type (midpoint sleep at ≥3:00). Further, the reproducibility of sleep duration assessments based on self-reporting was moderate (ICC = 0.572) (eTable 3).

**Table 1.** Baseline participant characteristics by sleep quality and duration status

	Total^a^ (*n* = 7,668)		Sleep quality and duration status												*P*-value

			SSD/SD (*n* = 701)		SSD/NSD (*n* = 100)		OSD/NSD (*n* = 1,863)		OSD/SD (*n* = 2,113)		LSD/NSD (*n* = 1,972)		LSD/SD (*n* = 919)		
Age, years^b^	73.3	(5.8)	72.7	(5.6)	70.4	(4.5)	71.2	(5.2)	73.0	(5.7)	74.4	(6.3)	76.5	(6.5)	<0.001
Women, *n* (%)^c^	4,017	(52.4)	427	(60.9)	66	(66.0)	1,019	(54.7)	1,184	(56.0)	840	(42.6)	481	(52.3)	<0.001
PD ≥1,000 people/km^2^, *n* (%)^c^	3,542	(46.2)	326	(46.5)	51	(51.0)	891	(47.8)	952	(45.1)	921	(46.7)	401	(43.6)	0.245
Body mass index, kg/m^2 b^	22.7	(3.4)	22.8	(4.2)	23.5	(4.8)	22.8	(3.1)	22.5	(3.1)	22.7	(3.5)	22.5	(3.9)	0.043
Living alone, *n* (%)^c^	882	(11.5)	103	(14.7)	8	(8.0)	196	(10.5)	268	(12.7)	190	(9.6)	117	(12.7)	<0.001
HSES, *n* (%)^c^	2,619	(34.2)	160	(22.8)	29	(29.0)	724	(38.9)	676	(32.0)	765	(38.8)	265	(28.8)	<0.001
Education ≥13 years, *n* (%)^c^	1,718	(22.4)	143	(20.4)	23	(23.0)	478	(25.7)	462	(21.9)	458	(23.2)	154	(16.8)	<0.001
Current smoker, *n* (%)^c^	808	(10.5)	56	(8.0)	8	(8.0)	193	(10.4)	208	(9.8)	248	(12.6)	95	(10.3)	<0.001
Alcohol drinker, *n* (%)^c^	5,037	(65.7)	434	(61.9)	60	(60.0)	1,238	(66.5)	1,377	(65.2)	1,351	(68.5)	577	(62.8)	0.005
Physical activity, MET-min/week^b^	774	(1,577)	672	(1,424)	1,124	(2,180)	876	(1,660)	724	(1,429)	839	(1,707)	584	(1,472)	<0.001
No medication, *n* (%)^c^	1,715	(22.4)	132	(18.8)	32	(32.0)	571	(30.7)	404	(19.1)	455	(23.1)	121	(13.2)	<0.001
Hypertension, *n* (%)^c^	2,902	(37.9)	274	(39.1)	34	(34.0)	617	(33.1)	833	(39.4)	757	(38.4)	387	(42.1)	<0.001
Stroke, *n* (%)^c^	271	(3.5)	29	(4.1)	3	(3.0)	39	(2.1)	73	(3.5)	84	(4.3)	43	(4.7)	0.002
Heart disease, *n* (%)^c^	932	(12.2)	97	(13.8)	7	(7.0)	168	(9.0)	290	(13.7)	212	(10.8)	158	(17.2)	<0.001
Diabetes, *n* (%)^c^	765	(10.0)	69	(9.8)	11	(11.0)	158	(8.5)	217	(10.3)	210	(10.7)	100	(10.9)	0.236
Hyperlipidemia, *n* (%)^c^	749	(9.8)	80	(11.4)	2	(2.0)	187	(10.0)	260	(12.3)	153	(7.8)	67	(7.3)	<0.001
Digestive disease, *n* (%)^c^	348	(4.5)	38	(5.4)	1	(1.0)	57	(3.1)	114	(5.4)	81	(4.1)	57	(6.2)	<0.001
Respiratory disease, *n* (%)^c^	639	(8.3)	81	(11.6)	2	(2.0)	104	(5.6)	217	(10.3)	154	(7.8)	81	(8.8)	<0.001
Urological diseases, *n* (%)^c^	467	(6.1)	47	(6.7)	1	(1.0)	94	(5.1)	142	(6.7)	113	(5.7)	70	(7.6)	0.014
Cancer, *n* (%)^c^	248	(3.2)	26	(3.7)	3	(3.0)	34	(1.8)	76	(3.6)	63	(3.2)	46	(5.0)	<0.001
Number of chronic diseases^b,d^	0.95	(0.97)	1.06	(1.03)	0.64	(0.77)	0.78	(0.90)	1.05	(1.01)	0.93	(0.96)	1.10	(1.00)	<0.001
Sleep duration, min/day^b^	435	(40)	291	(38)	317	(21)	413	(31)	398	(30)	513	(46)	524	(61)	<0.001
PSQI score^b^	6.0	(1.9)	11.1	(3.2)	4.3	(0.9)	3.6	(1.2)	8.3	(2.2)	3.1	(1.3)	7.7	(1.8)	<0.001

HSES, high socioeconomic status; LSD, long sleep duration; MET, metabolic equivalent; NSD, non-sleep disturbance; OSD, optimal sleep duration; PD, population density; PSQI, Pittsburgh Sleep Quality Index; SD, sleep disturbance; SSD, short sleep duration.

^a^Data for participants with missing values were imputed by multiple imputation: family structure (*n* = 558, 7.3%); socioeconomic status (*n* = 352, 4.6%); education (*n* = 826, 10.8%); smoking status (*n* = 323, 4.2%); alcohol status (*n* = 278, 3.6%); physical activity (*n* = 207, 2.7%); medications (*n* = 580, 7.6%).

^b^Continuous variables are expressed as mean with standard deviation and were analyzed using variance analysis.

^c^Category variables are expressed as number of cases with percentage and were analyzed using the Chi-square test.

^d^From the data obtained on disease status (including the presence of hypertension, stroke, heart disease, diabetes, hyperlipidemia, digestive disease, respiratory disease, urological diseases, and cancer), the comorbidity scores were summed to obtain a total score ranging from 0 (no comorbidity) to 9 (poor status).

### Association of sleep quality and duration with mortality risk

Table 2 shows the relationships of sleep quality and duration time with all-cause mortality risk. The median follow-up period was 4.75 years (34,826 person-years). During the follow-up period, 616 people (8.0%) died. After adjusting for confounders, SSD/SD and LSD/SD groups had the highest all-cause mortality risk compared with other groups (SSD/SD group: HR 1.56; 95% CI, 1.10–2.19; SSD/NSD group: HR 1.27; 95% CI, 0.47–3.48; OSD/NSD group: reference; OSD/SD group: HR 1.20; 95% CI, 0.91–1.59; LSD/NSD group: HR 1.35; 95% CI, 1.03–1.77; LSD/SD group: HR 1.83; 95% CI, 1.37–2.45). However, mortality risk was not associated with interaction between sleep quality and duration. Similar results were obtained in the sensitivity analysis (eTable 4, eTable 5, and eTable 6). The Nelson-Aalen cumulative hazard curves, where age was used as the time scale, showed a tendency toward larger differences in mortality hazard based on sleep quality and duration status with increasing age (eFigure 1).

**Table 2.** Hazard ratios for sleep quality and duration status and all-cause mortality calculated using multivariate Cox proportional hazards regression model

	*n*	Event	PY	Event/1,000 PY		Crude		Model 1^a^		Model 2^b^	

				Rate	95% CI	HR	95% CI	HR	95% CI	HR	95% CI
**Sleep quality × duration**											
SSD/SD	701	59	3,165	18.6	(14.4–24.1)	2.07	(1.47–2.90)	1.74	(1.24–2.45)	1.56	(1.10–2.19)
SSD/NSD	100	4	463	8.6	(3.2–23.0)	0.95	(0.35–2.61)	1.20	(0.44–3.29)	1.27	(0.47–3.48)
OSD/NSD	1,863	78	8,624	9.0	(7.2–11.3)	1.00	(Ref)	1.00	(Ref)	1.00	(Ref)
OSD/SD	2,113	147	9,636	15.3	(13.0–17.9)	1.69	(1.28–2.22)	1.35	(1.02–1.78)	1.20	(0.91–1.59)
LSD/NSD	1,972	181	8,942	20.2	(17.5–23.4)	2.24	(1.72–2.93)	1.40	(1.07–1.83)	1.35	(1.03–1.77)
LSD/SD	919	147	3,996	36.8	(31.3–43.2)	4.10	(3.12–5.40)	2.13	(1.60–2.84)	1.83	(1.37–2.45)
*Additive Interaction* ^c^											
SSD/SD						0.42	(39.7%)	0.19	(25.8%)	0.08	(14.1%)
LSD/SD						1.17	(37.7%)	0.39	(34.2%)	0.28	(33.7%)
*Multiplicative Interaction* ^d^											
SSD/SD						1.28	(0.45–3.66)	1.07	(0.38–3.07)	1.01	(0.36–2.90)
*P*-value						0.642		0.893		0.978	
LSD/SD						1.08	(0.76–1.54)	1.13	(0.80–1.61)	1.13	(0.79–1.60)
*P*-value						0.660		0.487		0.498	
**Sleep quality**											
NSD	3,935	263	18,030	14.6	(12.9–16.5)	1.00	(Ref)	1.00	(Ref)	1.00	(Ref)
SD	3,733	353	16,797	21.0	(18.9–23.3)	1.44	(1.23–1.69)	1.33	(1.13–1.56)	1.19	(1.01–1.40)
1 point increment						1.05	(1.03–1.07)	1.04	(1.02–1.07)	1.02	(1.00–1.05)
*P* for trend^2^						<0.001		<0.001		0.045	
**Sleep duration**											
SSD, <360 min/day	801	63	3,628	17.4	(13.6–22.2)	1.41	(1.07–1.87)	1.41	(1.06–1.86)	1.36	(1.02–1.79)
OSD, 360–480 min/day	3,976	225	18,260	12.3	(10.8–14.0)	1.00	(Ref)	1.00	(Ref)	1.00	(Ref)
LSD, >480 min/day	2,891	328	12,938	25.4	(22.8–28.2)	2.07	(1.74–2.45)	1.35	(1.13–1.61)	1.35	(1.13–1.60)

CI, confidence interval; HR, hazard ratio; LSD, long sleep duration; NSD, non-sleep disturbance; OSD, optimal sleep duration; PY, person-years; RERI, Relative Excess Risk due to Interaction; SD, sleep disturbance; SSD, short sleep duration.

^a^Model 1: Adjusted for age, sex, and population density.

^b^Model 2: In addition to the factors listed in model 1, adjusted for body mass index, living alone, socioeconomic status, educational attainment, smoking status, alcohol drinker, physical activity, medication use, and number of chronic diseases.

^c^The additive interaction was calculated as the RERI using the following equation: RERI [SSD/SD] = HR [SSD/SD] − (HR [SSD/NSD] + HR [OSD/SD] − 1) and RERI [LSD/SD] = HR [LSD/SD] − (HR [OSD/SD] + HR [LSD/NSD] − 1). The values are shown as RERI (%).

^d^It is significant (*P* < 0.05) if the 95% CI of the multiplicative interaction is not below 1.00.

### Dose-response relationship

**Figure 2.**
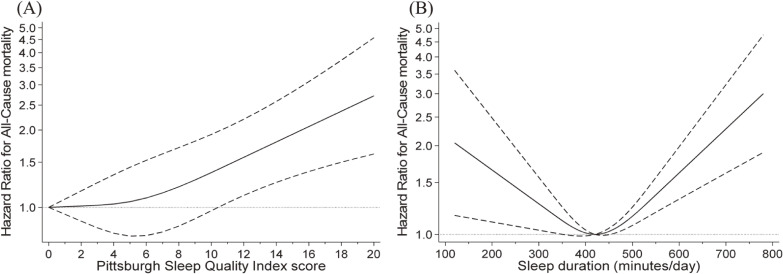
Restricted cubic spline model between sleep quality and duration and risk of mortality. Solid lines represent hazard ratios, and dashed lines represent 95% confidence intervals (CI), and the hazard ratio based on (**A**) 0 point for Pittsburgh Sleep Quality Index score and (**B**) 420 minutes/day for sleep duration as reference was calculated. We estimated that *P* ≥ 0.05 when the 95% CI of the hazard ratio did not exceed 1.00, and *P* < 0.05 when the 95% CI of the hazard ratio exceeded 1.00. The adjustment factors are age, sex, population density, body mass index, living alone, socioeconomic status, educational attainment, smoking status, alcohol drinker, physical activity, medication use, and number of chronic diseases.

Restricted cubic spline model was used to assess the curve relationships of sleep quality and duration to all-cause mortality risk (Figure 2). With a PSQI score of 0 as the reference, the curve was initially nearly flat up to a PSQI score of 5 but thereafter became a strongly positive log-linear relationship with all-cause mortality risk in a dose-dependent manner. The risk of mortality was significantly higher when PSQI exceeded an approximate score of 11 (reference: those with PSQI score of 0). Further, when sleep duration of 420 min/day was considered the reference, mortality risk and sleep duration exhibited a U-shaped relationship. Table 3 shows the relationships between PSQI subdomains and all-cause mortality. In all sleep duration subgroups, the daytime dysfunction score exhibited a positive association with all-cause mortality risk.

**Table 3.** Hazard ratios for the scores of the PSQI subdomains and all-cause mortality calculated using multivariate Cox proportional hazards regression model

Subdomain score	1 points increment					
	Model 1^a^			Model 2^b^		

	HR	95% CI	*P* for trend	HR	95% CI	*P* for trend
**Total (*n* = 7,667)**						
*Sleep quality*	1.18	(1.07–1.31)	0.001	1.11	(1.00–1.23)	0.041
*Sleep latency*	1.12	(1.03–1.21)	0.007	1.08	(0.99–1.16)	0.075
*Habitual sleep efficiency*	0.97	(0.90–1.05)	0.497	0.96	(0.88–1.04)	0.283
*Sleep disturbances*	1.29	(1.11–1.50)	0.001	1.20	(1.03–1.40)	0.020
*Medications to sleep*	1.13	(1.06–1.20)	<0.001	1.05	(0.99–1.13)	0.115
*Daytime dysfunction*	1.29	(1.16–1.45)	<0.001	1.24	(1.10–1.39)	<0.001
**Sleep duration <360 min (*n* = 801)**						
*Sleep quality*	1.34	(0.99–1.79)	0.051	1.21	(0.90–1.65)	0.211
*Sleep latency*	1.30	(1.01–1.66)	0.038	1.23	(0.95–1.58)	0.110
*Habitual sleep efficiency*	1.01	(0.81–1.26)	0.937	1.00	(0.80–1.24)	0.969
*Sleep disturbances*	1.87	(1.25–2.80)	0.002	1.70	(1.12–2.60)	0.014
*Medications to sleep*	1.09	(0.90–1.33)	0.387	1.04	(0.85–1.27)	0.720
*Daytime dysfunction*	1.48	(1.08–2.03)	0.015	1.48	(1.06–2.06)	0.020
**Sleep duration 360**–**480 min (*n* = 3,976)**						
*Sleep quality*	1.11	(0.92–1.33)	0.265	1.08	(0.89–1.30)	0.431
*Sleep latency*	1.08	(0.95–1.24)	0.235	1.06	(0.92–1.21)	0.436
*Habitual sleep efficiency*	0.91	(0.79–1.04)	0.158	0.91	(0.79–1.04)	0.163
*Sleep disturbances*	1.04	(0.79–1.36)	0.779	1.02	(0.78–1.34)	0.899
*Medications to sleep*	1.12	(1.01–1.25)	0.039	1.05	(0.94–1.18)	0.347
*Daytime dysfunction*	1.24	(1.03–1.50)	0.025	1.22	(1.00–1.48)	0.045
**Sleep duration >480 min (*n* = 2,890)**						
*Sleep quality*	1.27	(1.09–1.47)	0.002	1.18	(1.02–1.37)	0.027
*Sleep latency*	1.13	(1.01–1.27)	0.027	1.09	(0.98–1.22)	0.125
*Habitual sleep efficiency*	1.12	(0.95–1.33)	0.172	1.08	(0.92–1.28)	0.347
*Sleep disturbances*	1.35	(1.09–1.67)	0.006	1.24	(1.00–1.54)	0.049
*Medications to sleep*	1.15	(1.05–1.26)	0.002	1.07	(0.98–1.18)	0.128
*Daytime dysfunction*	1.31	(1.12–1.54)	0.001	1.22	(1.04–1.44)	0.016

CI, confidence interval; HR, hazard ratio; PSQI, Pittsburgh Sleep Quality Index.

The results are expressed as HR (95% CI) according to the 1-point increase in scores of the PSQI subdomains.

^a^Model 1: Adjusted for age, sex, and population density.

^b^Model 2: In addition to the factors listed in model 1, adjusted for body mass index, living alone, socioeconomic status, educational attainment, smoking status, alcohol drinker, physical activity, medication use, and number of chronic diseases.

## DISCUSSION

### Main findings

This study investigated how combinations of sleep quality and duration related to all-cause mortality in a population-based cohort of older people. SSD/SD and LSD/SD groups had the strongest associations with all-cause mortality risk compared with the other groups. However, mortality risk was not associated with the interaction between sleep quality and duration. Furthermore, with PSQI score of 0 as the reference, the relationship with all-cause mortality was nearly flat up to a PSQI score of 5 but thereafter became strongly positive log-linear relationship in a dose-dependent manner. When sleep duration of 420 min/day was considered the reference, mortality risk and sleep duration exhibited a U-shaped relationship. To the best of our knowledge, this is the first study to examine the association between mortality risk and combinations of sleep quality and duration in older Japanese adults assessed with a questionnaire composed of multidimensional sleep domains that have been previously validated. These findings suggest that both quality and duration of sleep should be evaluated in older people, as the adverse effects of sleep disorders on their all-cause mortality risk are dependent on their sleep duration.

### Prevalence of sleep disturbance

In this study, the SD prevalence evaluated with PSQI in older Japanese adults was 48.7%. The mean PSQI score was 6.0, similar to findings in a meta-analysis of older Chinese adults with a score of 6.64 points (95% CI, 6.14–7.13).^26^ This meta-analysis also showed that the proportion of sleep disturbances was higher in women than in men and in rural areas than in urban populations,^26^ consistent with our findings. As for chronotypes evaluated from the midpoint sleep time, the rate of SD was higher with the night type than with the morning type. People with sleep duration of <360 min/day and >480 min/day were found to have higher rates of SD.^21^ Findings from these previous studies appear to support that of the present study. Therefore, as the rate of SD is higher among the older adults,^4^ further studies are warranted to examine the relationship between sleep status and prognosis in community-dwelling older people in detail.

### Sleep quality and duration combinations and mortality risk

Our results showed a strong positive association between all-cause mortality risk and people with sleep disorders with both SSD and LSD. However, mortality risk was not associated with the interaction between sleep quality and duration. In a cohort study of middle-aged people aged 35–55 years, those who slept <6 hours and had poor sleep quality evaluated with a single domain had the highest risk of CVD mortality, but the study did not confirm multiple interactions of sleep quality and duration with the CVD mortality risk.^11^ This finding is similar to the results of this study, suggesting that the association of higher mortality risk with combinations of sleep quality and duration is additive rather than an interaction of these factors. In addition, people with poor sleep quality, sleeping <360 min or >480 min, reportedly have a higher prevalence of metabolic syndrome than those who sleep 7 hours with good sleep quality.^21^ Furthermore, people with multidimensional poor sleep quality with SSD, as evaluated with PSQI, are at the highest risk of developing CVD.^13^ These findings from previous studies support the association between high mortality risk and combinations of sleep quality and duration demonstrated in our study. As such, our findings suggest that combining the quality and duration of older individual’s sleep may help identify people who are at higher risk of death. Objective daily sleep duration, as evaluated using wearable devices, shows heterogeneity between countries and regions, with SSDs being strongly associated with later bedtimes than wake-up times.^27^ Many people go to bed late for a variety of work- and hobby-related reasons in modern societies; thus, our findings may provide further evidence for the benefits of improving sleep quality and duration, and provide data for future public health recommendations on sleep.

### Dose-response relationship

We observed a U-shaped relationship between sleep duration and mortality risk, with approximately 420 minutes of sleep per day having the lowest risk of death. This resembles the results of a meta-analysis^9^ and a pooled analysis^10^ that evaluated the relationship between sleep duration and mortality risk. Previous research examined the relationship between mortality risk and PSQI score by converting the continuous variable of the PSQI score into a categorical variable using a cut-off value.^13^ Our results indicated that PSQI scores ≥5.5, which were assessed as having SD, exhibited a strong log-linear relationship with all-cause mortality in a dose-dependent manner. Hence, our findings suggest that when the PSQI score is used to predict prognosis in the older adults, the relationship between their sleep status and mortality risk could be more accurately assessed using the PSQI score as a continuous variable rather than separating people into two groups using a cut-off value. In addition, findings on the relationship between PSQI subdomains and mortality risk may provide important insights into the sleep disorder risk factors that need to be resolved based on sleep duration.

### Mechanisms of sleep and death

While the detailed mechanism of why SSD associates positively with mortality risk remains unclear, previous research suggested three possible reasons.^9^^,^^10^^,^^28^ First, restricting nighttime sleep affects endocrine hormones, such as decreasing secretion of melatonin^29^ and testosterone.^30^ As shown, these endocrine abnormalities are associated with mortality^31^ and CVD onset.^31^^,^^32^ Second, shorter sleep durations increase appetite due to decreased leptin levels and increased ghrelin levels, and are associated with higher BMI.^33^ This supports the association with CVD onset in people with short sleep durations,^13^ and may partly explain the relationship between short sleep duration and mortality risk in the present study. Finally, having insufficient or irregular sleep may be associated with deteriorated risk factors for CVD due to a misalignment of the circadian rhythm.^34^ These results appear to support our finding of the strongest positive association with mortality risk in people with inadequate sleep quality and duration.

Furthermore, the underlying mechanism of the relationship between LSD and mortality risk may involve elevated levels of inflammatory markers, such as interleukin-6 and C-reactive protein.^35^ In addition, obstructive sleep apnea is characterized by long sleep duration, sleep disturbance, and daytime dysfunction and is also associated with increased risk of mortality and CVD.^36^ Thus, obstructive sleep apnea can mediate long sleep duration and mortality risk. Our results suggest that sleep quality (C1), sleep disturbances (C5), and daytime dysfunction (C7), which are features of obstructive sleep apnea, may predict mortality more than the other subdomain components in participants with LSD. Further studies are warranted for detailed examination of the relationship between the types of sleep disturbance and prognosis in community-dwelling older people. In contrast, such relationships may be due to residual confounding and pre-existing disease effects.^28^ LSDs are associated with various individual characteristics, including socioeconomic status, educational attainment, and comorbidities.^37^ As previously noted, association between long sleep duration and adverse outcomes may be related to existing causes that prolong sleep, particularly chronic diseases, which are likely to have a confounding effect.^38^ Although we adjusted for these confounding factors, it is not possible to prove a causal relationship between sleep status and mortality risk. Therefore, these relationships and their mechanisms need to be studied in more detail through interventional studies and basic research.

### Strengths and limitations

The strengths of this study are the evaluation of how combinations of sleep quality and duration relate to mortality using the validated PSQI^20^ in a large cohort of community-dwelling older adults. Our results may be more accurate and generalizable because the PSQI is a highly versatile method that has been validated in many countries.^7^ However, this study has methodological limitations. First, we could not evaluate the types of sleep disturbance in detail, including obstructive sleep apnea and restless legs syndrome. In addition, the self-reported sleep assessments may contain systematic reporting bias.^39^ Nonetheless, our findings were similar to those of a study that evaluated the association between objective sleep duration and mortality risk.^40^ We also showed that the reproducibility of sleep duration assessments based on self-reporting was moderate in this older cohort population; thus, the evaluations of sleep duration were likely to be relatively stable. Second, as the sleep status assessments were conducted only at baseline, participants’ sleep status may have changed during the follow-up period. Therefore, the association of the trajectory of age-related sleep status changes^41^ with mortality risk will henceforth need to be evaluated in older adults. Third, because data on the causes of death could not be obtained, we could not investigate how sleep quality and duration were related to the different causes of death. Further, our study had a relatively short follow-up period. Studies with shorter follow-ups have stronger associations between exposure variables and mortality risk than studies with longer follow-ups.^42^ This is because HRs estimated from an analysis may change over time, and results estimated from a shorter follow-up period may overestimate the relationship between exposure factors and outcomes or indicate causal reversal.^42^ However, we confirmed proportional hazards for the relationship between exposure and mortality risk, and similar results were obtained in a sensitivity analysis that excluded deaths occurring within the first 2 years of follow-up. Finally, although our study adjusted for confounding factors, residual confounding in the associations between sleep quality and duration with all-cause mortality risk may remain. Also, we were unable to assess the PSQI for all residents. Because the study participants were willing to complete the questionnaires, they may have been more health-conscious than the general older population, thereby creating possible selection bias. Therefore, we need objective assessments of sleep quality and duration and to re-evaluate our results in prospective studies with larger sample sizes and longer follow-up periods.

In conclusion, older adults with sleep disorders involving both SSD and LSD exhibited a strong positive association with all-cause mortality. This suggests that instead of an interaction between sleep quality and duration, there may be an additive effect. Furthermore, PSQI scores ≥5.5, which were assessed as having SD, exhibited a strong log-linear relationship with all-cause mortality in a dose-dependent manner. A U-shaped relationship was observed between sleep duration and mortality risk, with sleep of approximately 420 min/day having the lowest risk of death. These findings suggest that both the quality and duration of sleep need to be evaluated in older adults, as the adverse effects of the SD on their all-cause mortality risk depend on their sleep duration.

## SUPPLEMENTARY MATERIAL

eTable 1. Characteristics of participants at baseline and additional survey in the Kyoto-Kameoka Study

eTable 2. Prevalence rates of sleep disturbance defined by Pittsburgh Sleep Quality Index in older Japanese adults

eTable 3. Reproducibility of self-reported sleep duration

eTable 4. Results of sensitivity analysis for the relationship between sleep quality and duration status and all-cause mortality after excluding participants with an event in the first 2 years of follow-up

eTable 5. Results of sensitivity analysis for the relationship between sleep disturbance and tertile of sleep duration and all-cause mortality

eTable 6. Results of sensitivity analysis for the relationship between sleep quality and duration status and all-cause mortality after excluding participants with history of cardiovascular disease and cancer

eFigure 1. Nelson–Aalen Cumulative Hazard curves for all-cause mortality using age as the time scale according to sleep quality and duration status among older adults. (**A**) six groups stratified by sleep quality and duration: short sleep duration (SSD: < 360 min/day)/sleep disturbance (SD: ≥5.5 points of PSQI) group; SSD/non-sleep duration (NSD: SD: <5.5 points of PSQI) group; optimal sleep duration (OSD: 360–480 min/day)/NSD group; OSD/SD group; long sleep duration (LSD: > 480 min/day)/NSD group; LSD/SD group; (**B**) two groups stratified by sleep quality; (**C**) three groups stratified by sleep duration. PSQI, Pittsburgh Sleep Quality Index.
