# Supplementary material for: Combined Use of Sleep Quality and Duration Is More Closely Associated With Mortality Risk Among Older Adults: A Population-based Kyoto-Kameoka Prospective Cohort Study
Source: J Epidemiol. 2023 Dec 5;33(12):591–9. doi: 10.2188/jea.JE20220215 (PMC10635816; doi:10.2188/jea.JE20220215)
Supplement: Supplementary file 1 [file je-33-591-s001.pdf]

eTable 1. Characteristics of participants at baseline and additional survey in the Kyoto-Kameoka Study

	First survey[a]		First and second survey			
	All participants (n=13,294)		All participants (n=8,319)		Included participants (n=7,668)	
Age, years[b]	74.5	(6.9)	73.6	(6.2)	73.3	(5.8)
Women, n (%)[c]	7,337	(55.2)	4,412	(53.0)	4,017	(52.4)
PD ≥1,000 people/km^2, n (%)[c]	5,917	(44.5)	3,814	(45.8)	3,542	(46.2)
Body mass index, kg/m^2[b]	22.5	(3.6)	22.6	(3.5)	22.7	(3.4)
Living alone, n (%)[c]	1,695	(12.8)	966	(11.6)	882	(11.5)
HSES, n (%)[c]	4,228	(31.8)	2,770	(33.3)	2,619	(34.2)
Education ≥13 years, n (%)[c]	2,567	(19.3)	1,745	(21.0)	1,718	(22.4)
Current smoker, n (%)[c]	1,397	(10.5)	867	(10.4)	808	(10.5)
Alcohol drinker, n (%)[c]	8,277	(62.3)	5,470	(65.8)	5,037	(65.7)
Physical activity, MET-min/week[b]	663	(1470)	776	(1588)	774	(1577)
No medication, n (%)[c]	2,604	(19.6)	1,826	(21.9)	1,715	(22.4)
Hypertension, n (%)[c]	4,903	(36.9)	3,120	(37.5)	2,902	(37.9)
Stroke, n (%)[c]	617	(4.6)	296	(3.6)	271	(3.5)
Heart disease, n (%)[c]	1,659	(12.5)	1,016	(12.2)	932	(12.2)
Diabetes, n (%)[c]	1,390	(10.5)	838	(10.1)	765	(10.0)
Hyperlipidemia, n (%)[c]	1,122	(8.4)	780	(9.4)	749	(9.8)
Digestive disease, n (%)[c]	667	(5.0)	384	(4.6)	348	(4.5)
Respiratory disease, n (%)[c]	1,043	(7.8)	676	(8.1)	639	(8.3)
Urological diseases, n (%)[c]	817	(6.1)	494	(5.9)	467	(6.1)
Cancer, n (%)[c]	487	(3.7)	261	(3.1)	248	(3.2)
Number of chronic diseases[b, d]	0.96	(1.00)	0.95	(0.97)	0.95	(0.97)

HSES, high socioeconomic status; MET, metabolic equivalent; PD, population density.

[a] Missing values were supplemented using the multivariate imputation method in all participants: body mass index (n=1,039; 7.8%), family structure (n=1119; 8.4%), socioeconomic status (n=730; 5.5%), education attainment (n=1,895; 14.3%), smoking status (n=702; 5.3%), alcohol drinker (n=604; 4.5%), denture use (n=405; 3.0%), medications (n=1,140; 8.6%), frailty status (n=1,722; 13.0%), and physical activity (n=554; 4.2%). Body mass index was calculated as body weight (kg) divided by height squared (m^2).

[b] Continuous values are shown as mean (standard deviation).

[c] Categorical values are shown as number (percentage).

[d] From the data obtained on disease status (including the presence of hypertension, stroke, heart disease, diabetes, hyperlipidemia, digestive disease, respiratory disease, urological diseases, and cancer), the comorbidity scores were summed to obtain a total score ranging from 0 (no comorbidity) to 9 (poor status).

eTable 2. Prevalence rates of sleep disturbance defined by Pittsburgh Sleep Quality Index in older Japanese adults

	Prevalence of sleep disturbance				
	n	case	%	95% CI	*P*-value[a]
**Total**	7,668	3,733	48.7	(47.6–49.8)	
**Sex**					
Men	3,651	1,641	44.9	(43.3–46.6)	<0.001
Women	4,017	2,092	52.1	(50.5–53.6)	
**Age**					
65–74 years	4,795	2,210	46.1	(44.7–47.5)	<0.001
≥75 years	2,873	1,523	53.0	(51.2–54.8)	
**Population density**					
<1,000 people/km^2	4,126	2,054	49.8	(48.2–51.3)	0.038
≥1,000 people/km^2	3,542	1,679	47.4	(45.7–49.1)	
**Midpoint sleep time**					
<2:00	1,707	766	44.9	(42.5–47.3)	
2:00–2:59	3,505	1,703	48.6	(46.9–50.3)	<0.001
≥3:00	2,456	1,264	51.5	(49.5–53.5)	
**Sleep duration**					
<360 min/day	801	701	87.5	(85.0–89.7)	
360–480 min/day	3,976	2,113	53.1	(51.6–54.7)	<0.001
>480 min/day	2,891	919	31.8	(30.1–33.5)	

CI, confidence interval.

The Pittsburgh Sleep Quality Index (PSQI) score ranged from 0 (best sleep quality) to 21 (worse sleep quality), and sleep disturbance was defined as ≥5.5 points of PSQI.

[a] Statistical analysis was performed using the Chi-square test.

eTable 3. Reproducibility of self-reported sleep duration

	First survey		Second survey		Mean difference (95% CI)		ICC
	Mean	SD	Mean	SD			
**Total (*n*=7,668)**	403	(81)	435	(82)	-33	(-34 to -31)	0.572
**Sex**							
Men (*n*=3,651)	414	(83)	446	(83)	-31	(-34 to -29)	0.592
Women (*n*=4,017)	392	(78)	426	(80)	-34	(-36 to -31)	0.538
**Age**							
65–74 years (*n*=4,795)	394	(73)	424	(73)	-29	(-32 to -27)	0.574
≥75 years (*n*=2,873)	418	(91)	455	(91)	-38	(-41 to -34)	0.533
**Sleep quality**							
Non-case (*n*=3,935)	422	(77)	461	(67)	-39	(-41 to -37)	0.570
Sleep disturbance (*n*=3,733)	383	(81)	409	(88)	-26	(-29 to -23)	0.520

CI, confidence intervals; ICC, intraclass correlation coefficient; SD, standard deviation.

The baseline (first) survey was conducted on 29 July 2011, and the additional (second) survey on February 14, 2012.

eTable 4. Results of sensitivity analysis for the relationship between sleep quality and duration status and all-cause mortality after excluding participants with an event in the first 2 years of follow-up

	n	Event	PY	Event/1,000 PY		Crude		Model 1[a]		Model 2[b]	
				Rate	95% CI	HR	95% CI	HR	95% CI	HR	95% CI
**Sleep quality×duration**											
SSD/SD	682	40	3,144	12.7	(9.3–17.3)	2.20	(1.45–3.34)	1.83	(1.20–2.77)	1.65	(1.09–2.52)
SSD/NSD	99	3	463	6.5	(2.1–20.1)	1.11	(0.35–3.57)	1.39	(0.43–4.46)	1.49	(0.46–4.78)
OSD/NSD	1,835	50	8,589	5.8	(4.4–7.7)	1.00	(Ref)	1.00	(Ref)	1.00	(Ref)
OSD/SD	2,066	100	9,585	10.4	(8.6–12.7)	1.80	(1.28–2.53)	1.42	(1.01–2.00)	1.28	(0.91–1.80)
LSD/NSD	1,920	129	8,885	14.5	(12.2–17.3)	2.52	(1.82–3.49)	1.57	(1.13–2.19)	1.52	(1.09–2.12)
LSD/SD	866	94	3,941	23.8	(19.5–29.2)	4.19	(2.97–5.90)	2.16	(1.52–3.08)	1.89	(1.32–2.71)
Additive Interaction[c]											
SSD/SD						0.29	(23.8%)	0.01	(1.3%)	-0.11	(-17.0%)
LSD/SD						0.87	(27.3%)	0.17	(14.3%)	0.09	(10.4%)
Multiplicative Interaction[d]											
SSD/SD						1.10	(0.32–3.72)	0.92	(0.27–3.13)	0.87	(0.26–2.95)
P-value							0.883		0.896		0.824
LSD/SD						0.92	(0.60–1.42)	0.97	(0.63–1.49)	0.97	(0.63–1.50)
P-value							0.717		0.875		0.902
**Sleep quality**											
Non-case	3,854	182	17,937	10.1	(8.8–11.7)	1.00	(Ref)	1.00	(Ref)	1.00	(Ref)
Sleep disturbance	3,614	234	16,670	14.0	(12.3–16.0)	1.39	(1.15–1.69)	1.27	(1.04–1.54)	1.14	(0.94–1.39)
1 points increment						1.03	(1.01–1.06)	1.02	(0.99–1.05)	1.01	(0.98–1.03)
P for trend							0.017		0.097		0.701
**Sleep duration**											
SSD, <360 min/day	781	43	3607	11.9	(8.8–16.1)	1.45	(1.03–2.04)	1.43	(1.02–2.01)	1.40	(1.00–1.96)
OSD, 360–480 min/day	3,901	150	18,174	8.3	(7.0–9.7)	1.00	(Ref)	1.00	(Ref)	1.00	(Ref)
LSD, >480 min/day	2,786	223	12,827	17.4	(15.2–19.8)	2.13	(1.73–2.62)	1.41	(1.14–1.74)	1.40	(1.13–1.74)

CI, confidence interval; HR, hazard ratio; LSD, long sleep duration; NSD, non-sleep disturbance; OSD, optimal sleep duration; PY, person-years; RERI, Relative Excess Risk due to Interaction; SD, sleep disturbance; SSD, short sleep duration.

[a] Model 1: Adjusted for age, sex, and population density.

[b] Model 2: In addition to the factors listed in model 1, adjusted for body mass index, living alone, socioeconomic status, educational attainment, smoking status, alcohol drinker, physical activity, medication use, and number of chronic diseases.

[c] The additive interaction was calculated as RERI using the following equation: RERI [SSD/SD] = HR [SSD/SD] – (HR [SSD/NSD] + HR [OSD/SD] –1) and RERI [LSD/SD] = HR [LSD/SD] – (HR [OSD/SD] + HR [LSD/NSD] –1). The values are shown as RERI (%).

[d] It is significant ($P<0.05$) if the 95% CI of the multiplicative interaction is not below 1.00.

eTable 5. Results of sensitivity analysis for the relationship between sleep disturbance and tertile of sleep duration and all-cause mortality

	n	Event	PY	Event/1,000 PY		Crude		Model 1[a]		Model 2[b]	
				Rate	95% CI	HR	95% CI	HR	95% CI	HR	95% CI
**Sleep quality×duration**											
T1/SD	1,043	75	4,739	15.8	(12.6–19.8)	1.53	(1.09–2.15)	1.22	(0.87–1.71)	1.01	(0.72–1.42)
T1/NSD	690	21	3,201	6.6	(4.3–10.1)	0.63	(0.39–1.04)	0.72	(0.44–1.18)	0.76	(0.46–1.25)
T2/NSD	1,273	61	5,887	10.4	(8.1–13.3)	1.00	(Ref)	1.00	(Ref)	1.00	(Ref)
T2/SD	1,771	131	8,062	16.2	(13.7–19.3)	1.57	(1.16–2.13)	1.35	(1.00–1.83)	1.13	(0.83–1.54)
T3/NSD	1,972	181	8,942	20.2	(17.5–23.4)	1.96	(1.47–2.62)	1.26	(0.94–1.69)	1.23	(0.91–1.65)
T3/SD	919	147	3,996	36.8	(31.3–43.2)	3.58	(2.66–4.83)	1.92	(1.42–2.62)	1.52	(1.11–2.08)
Additive Interaction[c]											
T1/SD						0.33	(61.5%)	0.15	(67.7%)	0.12	(1411.3%)
T3/SD						1.05	(40.7%)	0.31	(33.7%)	0.17	(31.9%)
Multiplicative Interaction[d]											
T1/SD						1.62	(0.92–2.87)	1.55	(0.88–2.75)	1.47	(0.83–2.60)
P-value							0.097		0.131		0.188
T3/SD						1.19	(0.80–1.79)	1.26	(0.84–1.88)	1.23	(0.82–1.84)
P-value							0.385		0.266		0.315
**Sleep duration**											
T1	2,461	152	11,263	13.5	(1.15–15.8)	1.05	(0.84–1.33)	1.09	(0.86–1.37)	1.05	(0.83–1.32)
T2	2,316	136	10,626	12.8	(10.8–15.1)	1.00	(Ref)	1.00	(Ref)	1.00	(Ref)
T3	2,891	328	12,938	25.4	(22.8–28.2)	1.99	(1.63–2.43)	1.32	(1.08–1.63)	1.32	(1.08–1.62)

CI, confidence interval; HR, hazard ratio; NSD, non-sleep disturbance; PY, person-years; RERI, Relative Excess Risk due to Interaction; SD, sleep disturbance; T, tertile.

T1, T2, and T3 include the sleep duration of <419 minutes/day, 420–475 minutes/day, and ≥480 minutes/day, respectively.

[a] Model 1: Adjusted for age, sex, and population density.

[b] Model 2: In addition to the factors listed in model 1, adjusted for body mass index, living alone, socioeconomic status, educational attainment, smoking status, alcohol drinker, physical activity, medication use, and number of chronic diseases.

[c] The additive interaction was calculated as RERI using the following equation: RERI [T1/SD] = HR [T1/SD] – (HR [T1/NSD] + HR [T2/SD] –1) and RERI [T3/SD] = HR [T3/SD] – (HR [T2/SD] + HR [T3/NSD] –1). The values are shown as RERI (%).

[d] It is significant (*P*<0.05) if the 95% CI of the multiplicative interaction is not below 1.00.

eTable 6. Results of sensitivity analysis for the relationship between sleep quality and duration status and all-cause mortality after excluding participants with history of cardiovascular disease and cancer

	n	Event	PY	Event/1,000 PY		Crude		Model 1[a]		Model 2[b]	
				Rate	95% CI	HR	95% CI	HR	95% CI	HR	95% CI
**Sleep quality×duration**											
SSD/SD	535	38	2,442	15.6	(11.3–21.4)	2.13	(1.41–3.22)	1.82	(1.20–2.76)	1.63	(1.07–2.49)
SSD/NSD	87	3	403	7.5	(2.4–23.1)	1.02	(0.32–3.25)	1.30	(0.41–4.16)	1.31	(0.41–4.20)
OSD/NSD	1,585	54	7,371	7.3	(5.6–9.6)	1.00	(Ref)	1.00	(Ref)	1.00	(Ref)
OSD/SD	1,628	90	7,473	12.0	(9.8–14.8)	1.65	(1.17–2.31)	1.32	(0.94–1.85)	1.20	(0.85–1.69)
LSD/NSD	1,587	113	7,249	15.6	(13.0–18.7)	2.13	(1.54–2.95)	1.32	(0.95–1.84)	1.30	(0.93–1.81)
LSD/SD	652	78	2,912	26.8	(21.5–33.4)	3.68	(2.60–5.21)	1.82	(1.27–2.61)	1.59	(1.10–2.30)
Additive Interaction[c]											
SSD/SD						0.47	(41.2%)	0.20	(24.5%)	0.12	(19.5%)
LSD/SD						0.90	(33.6%)	0.18	(21.7%)	0.09	(14.6%)
Multiplicative Interaction[d]											
SSD/SD						1.27	(0.37–4.32)	1.06	(0.31–3.61)	1.04	(0.31–3.54)
P-value							0.700		0.924		0.951
LSD/SD						1.05	(0.67–1.63)	1.04	(0.67–1.63)	1.02	(0.65–1.59)
P-value							0.835		0.853		0.943
**Sleep quality**											
Non-case	3,259	170	15,022	11.3	(9.7–13.2)	1.00	(Ref)	1.00	(Ref)	1.00	(Ref)
Sleep disturbance	2,815	206	12,827	16.1	(14.0–18.4)	1.42	(1.16–1.74)	1.30	(1.06–1.59)	1.17	(0.95–1.44)
1 points increment						1.05	(1.02–1.08)	1.04	(1.01–1.07)	1.02	(0.99–1.05)
P for trend							0.001		0.009		0.224
**Sleep duration**											
SSD, <360 min/day	622	41	2,845	14.4	(10.6–19.6)	1.49	(1.05–2.10)	1.50	(1.06–2.13)	1.43	(1.01–2.03)
OSD, 360–480 min/day	3,213	144	14,843	9.7	(8.2–11.4)	1.00	(Ref)	1.00	(Ref)	1.00	(Ref)
LSD, >480 min/day	2,239	191	10,161	18.8	(16.3–21.7)	1.94	(1.57–2.41)	1.24	(1.01–1.46)	1.25	(1.00–1.56)

CI, confidence interval; HR, hazard ratio; LSD, long sleep duration; NSD, non-sleep disturbance; OSD, optimal sleep duration; PY, person-years; RERI, Relative Excess Risk due to Interaction; SD, sleep disturbance; SSD, short sleep duration.

Cardiovascular disease was defined as stroke and heart disease.

[a] Model 1: Adjusted for age, sex, and population density.

[b] Model 2: In addition to the factors listed in model 1, adjusted for body mass index, living alone, socioeconomic status, educational attainment, smoking status, alcohol drinker, physical activity, medication use, and number of chronic diseases.

[c] The additive interaction was calculated as RERI using the following equation: RERI [SSD/SD] = HR [SSD/SD] – (HR [SSD/NSD] + HR [OSD/SD] –1) and RERI [LSD/SD] = HR [LSD/SD] – (HR [OSD/SD] + HR [LSD/NSD] –1). The values are shown as RERI (%).

[d] It is significant ($P<0.05$) if the 95% CI of the multiplicative interaction is not below 1.00.

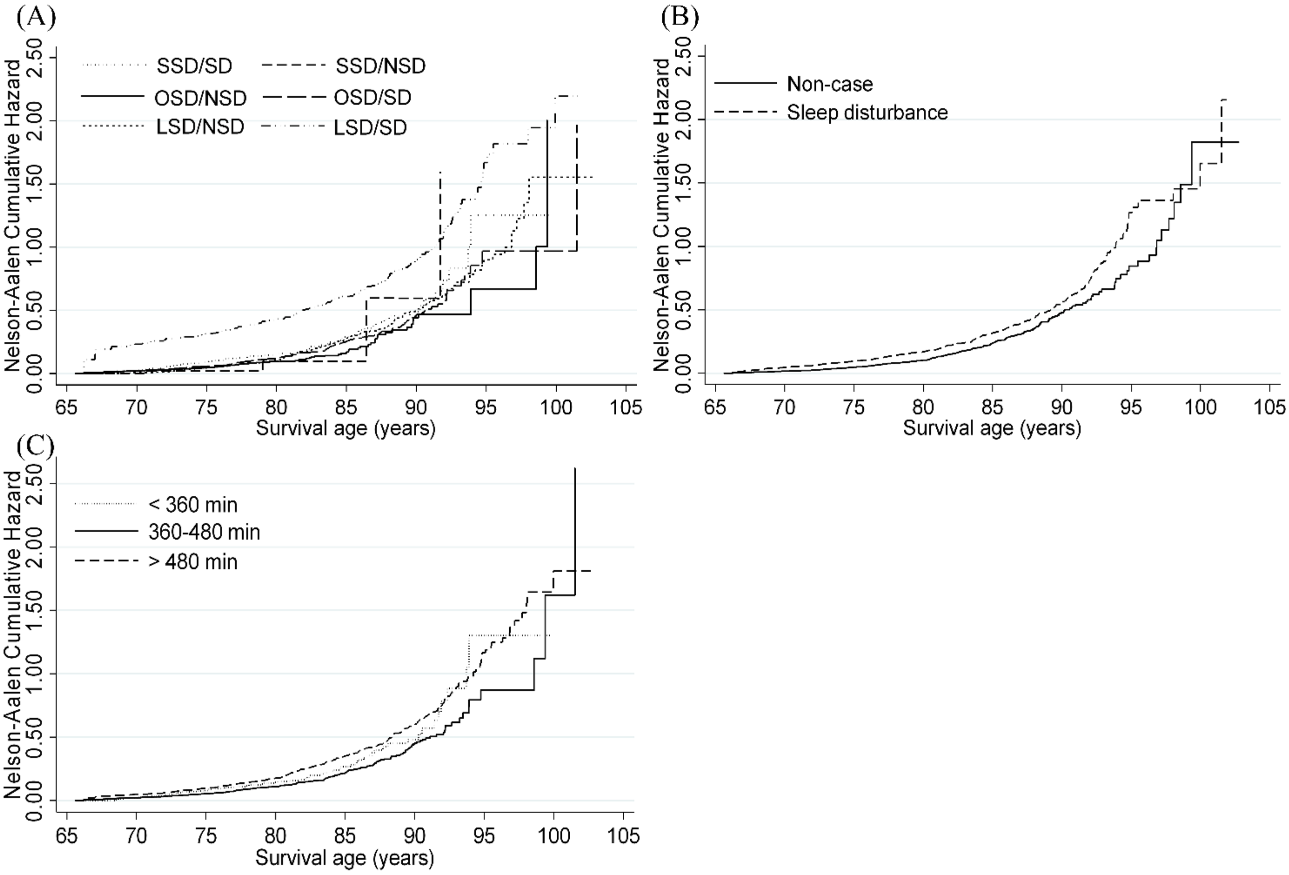

eFigure 1. Nelson–Aalen Cumulative Hazard curves for all-cause mortality using age as the time scale according to sleep quality and duration status among older adults. (**A**) six groups stratified by sleep quality and duration: short sleep duration (SSD: < 360 min/day)/sleep disturbance (SD: ≥5.5 points of PSQI) group; SSD/non-sleep duration (NSD: SD: <5.5 points of PSQI) group; optimal sleep duration (OSD: 360-480 min/day)/NSD group; OSD/SD group; long sleep duration (LSD: > 480 min/day)/NSD group; LSD/SD group; (**B**) two groups stratified by sleep quality; (**C**) three groups stratified by sleep duration. PSQI, Pittsburgh Sleep Quality Index.
